# Supplementary material for: A symmetrical method to obtain shear moduli from microrheology
Source: Soft Matter. 2018 Mar 21;14(19):3716–23. doi: 10.1039/c7sm02499a (PMC5954977; doi:10.1039/c7sm02499a)
Supplement: Supplementary file 1 [file SM-014-C7SM02499A-s001.pdf]

Electronic Supplementary Material (ESI) for Soft Matter.
This journal is © The Royal Society of Chemistry 2018

A Supplementary Information

A.1 Summary of method introduced by Evans et al.

The Evans et al. method is based on the following relation[22–24]

$$-i\omega 2kT\chi(\omega) \simeq \sum_{k=1}^{N}\left(\frac{M_k - M_{k-1}}{t_k - t_{k-1}}\right)\left[e^{i\omega t_{k-1}} - e^{i\omega t_k}\right] - i\omega M_0 + e^{i\omega t_N}\dot{M}(t_N), \quad (20)$$

where the right hand side approximates the Fourier transform of the second derivative of the MSD, which is sampled at times $t = 0, t_1, t_2, t_3, \ldots, t_N$, where the values are $M_0, M_1, M_2, M_3, \ldots, M_N$. This allows for a finite derivative $\dot{M}$ of the MSD at time t_N, as can be expected for a viscous fluid response at long times. For the present case of trapped probe particles, we assume that this derivative vanishes. Furthermore, we also assume that the MSD extrapolates continuously to $M_0 = 0$ at $t = 0$. The resulting expressions for real and imaginary parts of χ are:

$$\chi'(\omega) = \frac{-1}{2kT\omega}S''_M \quad (21)$$

and

$$\chi''(\omega) = \frac{1}{2kT\omega}S'_M, \quad (22)$$

where S'_M and S''_M are real and imaginary parts of

$$S_M(\omega) = \sum_{k=1}^{N}\left(\frac{M_k - M_{k-1}}{t_k - t_{k-1}}\right)\left[e^{i\omega t_{k-1}} - e^{i\omega t_k}\right]. \quad (23)$$

A.2 Algorithm for symmetric method

The following is the code used in IgorPro for the evaluation of the complex χ and K in Fig. 2 from discretely sampled values of the MSD with equal spacing in time. For general use the parameter `kBT` and data for the MSD and time series (`tau`) need to be supplied.

```
Function chiw_cal(m)

variable m // the number of data points of the response function in omega (chi(w))
variable k  // the number of data points
wave MSD,tau
variable i,j,l
variable num
variable kBT,dt

k=DimSize(MSD,0)
Make/O/N=(k) chit  // response function in time domain

dt=tau[1]-tau[0]
kBT=0.5
chit= 0

// numerical derivative

for (i=0; i< 2; i+=1)
chit[i]=1/12*(-25*MSD[i]+48*MSD[i+1]-36*MSD[i+2]+16*MSD[i+3]-3*MSD[i+4])/kBT/2/dt
endfor

for (i=2; i< k-2; i+=1)
chit[i]=1/12*(MSD[i-2]-8*MSD[i-1]+8*MSD[i+1]-MSD[i+2])/kBT/2/dt
endfor

for (i=k-2; i< k; i+=1)
chit[i]=1/2*(MSD[i-2]-4*MSD[i-1]+3*MSD[i])/kBT/2/dt
endfor

Make/O/N=(m) w0
Make/O/N=(m) chi1   // the real part of the response function
Make/O/N=(m) chi2   // the imaginary part of the response function

w0=(x+1)*pi/m/dt    // generate omega
```

```
chi1=0
chi2=0

l=(k-3)/2

// fourier transform

for (i=0; i< m; i+=1)

for (j=0; j<1 ; j+=1)
chi2[i] += chit[j]*sin(w0[i]*tau[j])/3*dt
chi1[i] += chit[j]*cos(w0[i]*tau[j])/3*dt
endfor

for(j=1;j < l+1;j+=1)
chi2[i] += chit[2*j-1]*sin(w0[i]*tau[2*j-1])*4/3*dt+chit[2*j]*sin(w0[i]*tau[2*j])*2/3*dt
chi1[i] += chit[2*j-1]*cos(w0[i]*tau[2*j-1])*4/3*dt+chit[2*j]*cos(w0[i]*tau[2*j])*2/3*dt
endfor

for(j=l+1;j < l+2;j+=1)
chi2[i] += chit[2*j-1]*sin(w0[i]*tau[2*j-1])*4/3*dt+chit[2*j]*sin(w0[i]*tau[2*j])/3*dt
chi1[i] += chit[2*j-1]*cos(w0[i]*tau[2*j-1])*4/3*dt+chit[2*j]*cos(w0[i]*tau[2*j])/3*dt
endfor

endfor

End
```

References

22 R. M. L. Evans, M. Tassieri, D. Auhl, and T. A. Waigh, Physical Review E **80**, 012501 (2009).

23 R. L. Evans, Br. Soc. Rheol. Bull. **50**, 76 (2009).

24 M. Tassieri, R. M. L. Evans, R. L. Warren, N. J. Bailey, and J. Cooper, New J. Phys. **14**, 115032 (2012).
